# Polyhydroxyurethane and Poly(ethylene oxide) Multiblock Copolymer Networks: Crosslinking with Polysilsesquioxane, Reprocessing and Solid Polyelectrolyte Properties

**DOI:** 10.3390/polym15244634

**Published:** 2023-12-07

**Authors:** Lei Li, Bingjie Zhao, Guohua Hang, Yuan Gao, Jiawei Hu, Tao Zhang, Sixun Zheng

**Affiliations:** Department of Polymer Science and Engineering and the State Key Laboratory of Metal Matrix Composites, Shanghai Jiao Tong University, Shanghai 200240, China

**Keywords:** polyhydroxyurethane, poly(ethylene oxide), networks, silyl ether bond metathesis, reprocessing properties, ionic conductivity

## Abstract

This contribution reports the synthesis of polyhydroxyurethane (PHU)-poly(ethylene oxide) (PEO) multiblock copolymer networks crosslinked with polysilsesquioxane (PSSQ). First, the linear PHU-PEO multiblock copolymers were synthesized via the step-growth polymerization of bis(6-membered cyclic carbonate) (B6CC) with α,ω-diamino-terminated PEOs with variable molecular weights. Thereafter, the PHU-PEO copolymers were allowed to react with 3-isocyanatopropyltriethoxysilane (IPTS) to afford the derivatives bearing triethoxysilane moieties, the hydrolysis and condensation of which afforded the PHU-PEO networks crosslinked with PSSQ. It was found that the PHU-PEO networks displayed excellent reprocessing properties in the presence of trifluoromethanesulfonate [Zn(OTf)_2_]. Compared to the PHU networks crosslinked via the reaction of difunctional cyclic carbonate with multifunctional amines, the organic–inorganic PHU networks displayed the decreased reprocessing temperature. The metathesis of silyl ether bonds is responsible for the improved reprocessing behavior. By adding lithium trifluoromethanesulfonate (LiOTf), the PHU-PEO networks were further transformed into the solid polymer electrolytes. It was found that the crystallization of PEO chains in the crosslinked networks was significantly suppressed. The solid polymer electrolytes had the ionic conductivity as high as 7.64 × 10^−5^ S × cm^−1^ at 300 K. More importantly, the solid polymer electrolytes were recyclable; the reprocessing did not affect the ionic conductivity.

## 1. Introduction

Polyurethanes (PUs) are a class of major synthetic polymers and have been applied in various fields [1,2,3,4,5]. In general, PUs are synthesized via the step-growth polymerization of diisocyanates with diols or/and polyols. In terms of the types and compositions of the reactants (or monomers), PUs can be designed to display the thermomechanical properties from elastomers, thermoplastics to thermosets. Albeit PUs have wide-spread applications, there has long been the concern about the synthesis of diisocyanates (i.e., one of the monomers) since the starting compound for diisocyanates (i.e., phosgene) is highly toxic [6,7,8]. Recently, it has been realized that isocyanates themselves are also harmful to human health [9,10]. Therefore, there is a high demand for the synthesis of PUs via so-called non-isocyanate routes or for finding PU substitutes. In the past years, a considerable effort has been made to solve this issue [11,12,13,14,15,16]. Poly(hydroxyurethane)s (PHUs) are among the promising alternatives to traditional PUs [17,18,19,20,21]. PHUs can be synthesized via the ring-opening polyaddition of cyclic carbonates with amines [22,23,24,25,26]. Likewise, PHUs can be formulated into thermoplastics and thermosets, thermoplastic elastomers, and coatings [27,28,29,30,31,32,33,34,35,36,37,38]. For example, Torkelson et al. [27,28,29,30,31] have reported the synthesis of various linear (viz. thermoplastic) PHUs. Mülhaupt et al. [39] reported the synthesis of bio-based PHU thermoplastics from biomass resources (e.g., erythritol, cinene). From the viewpoint of macromolecular structures, PHUs have a similar structure of main chains but carry a great number of primary or secondary hydroxyl groups. Owing to the presence of hydroxyl groups, PHUs have some additional properties such as resistance to nonpolar solvents and adhesive properties. In addition, the side hydroxyl groups can be further used for the modification of PHUs or post crosslinking.

Crosslinked PHUs have also attracted a considerable interest because of their improved solvent resistance, thermal stability and mechanical properties compared with linear PHUs. In marked contrast to traditional crosslinked polymers, crosslinked PHUs themselves can be reprocessed without introducing dynamic covalent bonds. The reprocessing properties are attributable to the dynamic exchange reactions of carbamate moieties with hydroxyl groups at elevated temperatures. Hillmyer and Dichtel et al. [40,41] reported that the PHU networks from a six-membered cyclic carbonate and multiple amine can be reprocessed at elevated temperature (i.e., 170 °C or higher) and the mechanical properties can be recovered to 75%. Based on the crosslinking of difunctional five-membered cyclic carbonates with polyamines, Torkelson et al. [42] investigated the accelerated reprocessing and improved mechanical recovery by introducing transcarbamoylation catalyst (i.e., 4-dimethylaminopyridine). More recently, Seychal et al. [43] synthesized five cyclic carbonates and the corresponding crosslinked PHUs. Under the catalyst-free conditions, it was also found that all PHU networks displayed the stress relax behavior through the transcarbamoylation reactions at elevated temperature. Nonetheless, the reprocessing behavior required sufficiently high temperature (i.e., 160 °C) and long time (i.e., 15 h). The thermal treatment at fairly high temperature and with a long time would give rise to thermal degradation, which is one of the causes that the PHU networks displayed the incomplete recovery of mechanical strengths. Recently, a considerable interest has been attracted to introduce new dynamic chemical bonds to improve the reprocessing properties of PHU networks. For instance, Torkelson et al. [44] reported the introduction of disulfide bonds into PHU networks; it was demonstrated that the PHU networks displayed improved reprocessing properties. More recently, Zheng et al. [45] reported the synthesis of PHU networks crosslinked with trifunctional polyhedral oligomeric silsesquixanes (POSS) via Diels–Alder (D–A) reaction. The introduction of D–A adducts allowed for the reprocessing of PHU-POSS networks to be performed at quite low temperature (i.e., 120 °C) for a short time (i.e., 20 min). In all previous reports, most of the PHU networks were obtained via the one-step crosslinking approach, i.e., the networks were generated with direct crosslinking of difunctional cyclic carbonates with polyamine. Compared with the direct crosslinking approach, the post crosslinking of PHU chains allows more flexible designs of network structures, which can endow the materials with new properties. Nonetheless, such an investigation remains largely unexplored.

In this contribution, we reported a facile approach to obtain PHU networks via the post crosslinking of linear PHU-PEO multiblock copolymers. Toward this end, a series of linear polyhydroxyurethane (PHU) and poly(ethylene oxide) multiblock copolymers were first synthesized via the polyaddition of bis(6-membered cyclic carbonate) (B6CC) with α,ω-diamino-terminated poly(ethylene oxide) (NH_2_-PEO-NH_2_). The as-obtained PHU-PEO copolymers were then reacted with the 3-isocyanatopropyltriethoxysilane (IPTS) to afford the PHU-PEO multiblock copolymers bearing triethoxysilane pendent groups. Upon hydrolyzing and condensing, the PHU-PEO copolymers were crosslinked with PSSQ segments. It is anticipated that the crosslinking of PHU with inorganic segments (i.e., PSSQ) would significantly improve the thermomechanical properties of the PHU networks. Owing to the generation of PSSQ linkages, sily ether bonds were simultaneously introduced to the PHU networks. It has been realized that silyl ether bonds can be transformed into dynamic covalent bonds while Lewis acid (e.g., trifluoromethanesulfonate, Zn(OTf)_2_) was added to the system [46]. With the introduction of new dynamic covalent bonds, the reprocessing temperature of the PHU networks would be significantly decreased [46,47,48,49,50]. In addition, the PHU-PEO-PSSQ networks are readily formulated into solid polymer electrolytes (SPEs) by introducing lithium trifluoromethanesulfonate (LiOTf) [46,47,48,49]. It is proposed that the introduction of the hydrophobic PSSQ segments would diminish the impact of moisture absorption on the electric conductivity. The ionic conductivity can be modulated according to the PEO contents in the networks. Furthermore, the networks of SPEs are recyclable with the build-in of two motifs (i.e., hydroxyl urethane structural units and sily ether bonds). Such reprocessing properties are important for the recycling of SPEs. The purpose of this work is two-fold: (i) to report an approach to obtain PHU networks in a post-crosslinking fashion and (ii) to decrease the reprocessing temperature by introducing the metathesis of sily ether bonds. 

## 2. Experimental

### 2.1. Materials

3-Isocyanatopropyltriethoxysilane, trifluoromethanesulfonate [Zn(OTf)_2_]_,_ lithium trifluoromethyl sulfonate (LiOTf), dibutyltindilaurate and hydrochloric acid were supplied by Adamas Reagent Co., Shanghai, China. Bis(6-membered cyclic carbonate) (B6CC) was synthesized by following the method of literature [50], as detailed in Supporting Information (SI). α,ω-Diamino-terminated poly(ethylene oxide)s (denoted NH_2_-PEO-NH_2_) with the molecular weights of *M*_n_ = 400, 600, 1000, 2000 and 4000) were synthesized in this lab, as detailed in Supporting Information (SI) [51].

### 2.2. Synthesis of PHU-PEO Multiblock Copolymers 

The linear PHU-PEO multiblock copolymers were synthesized via the step-growth polymerization of B6CC with NH_2_-PEO-NH_2_. Typically, B6CC (0.906 g, 0.003 mmol), NH_2_-PEO-NH_2_ (3.000 g, 0.003 mol, *M*_n_ = 1000 Da) and 1,4-dioxane (4 mL) were charged in a 25 mL flask. The system was purged with highly pure nitrogen for 30 min, and then the polymerization was conducted at 90 °C for 36 h. The viscous mixture was dropped into petroleum ether to afford the precipitates. After drying, the copolymer (denoted PHU-PEO_1000_) (3.850 g) was afforded with a 98.6% yield. ^1^H NMR (CDCl_3_, ppm): 4.22 (*m*, -C***H***_2_OC=O), 3.95 (*m*, -C***H***_2_OH), 3.56 (*m*, -C***H***_2_C***H***_2_O-), 3.22 (*m*, -C***H***_2_O-), 1.32 (*m*, -C***H***_2_CH_3_), 0.85 (*m*, -CH_2_C***H***_3_). GPC: *M*_n_ = 31,200 Da with *M*_w_/*M*_n_ = 1.45.

### 2.3. Synthesis of PHU-PEO-PSSQ Networks 

To a flask, PHU-PEO_1000_ (3.000 g) dissolved in DMF (3 mL) and 3-isocyanatopropyltriethoxysilane (0.797 g, 3.33 mmol) were added. A catalytic amount of zinc trifluoromethanesulfonate [Zn(OTf)_2_] (i.e., 5 wt% with respect of the overall mass of the reactants) and dibutyltindilaurate (50 uL) were added in turn. The reaction was conducted at 70 °C for 10 h. Thereafter, a small amount of diluted hydrochloric acid was added. Notably, the mixture gradually gelled. After it dried at 60 °C for 24 h, and then at 40 °C in vacuo for 12 h, the gel was chopped into small pieces. Further dried at 40 °C in vacuo to the constant weight, the as-obtained product (denoted PHU-PEO_1000_-PSSQ network) was obtained for the subsequent measurements. 

### 2.4. Preparation of Solid Polymer Electrolytes (PHU-PEO-LiOTf)

Typically, PHU-PEO_1000_ (3.000 g) dissolved in DMF (3 mL) and 3-isocyanatopropyltriethoxysilane (0.797 g, 3.22 mmol) were added to a flask with vigorous stirring. Thereafter, Zn(OTf)_2_ (5 wt% with respect of the overall mass of the reactants), LiOTf (0.739 g, 4.74 mmol) and dibutyltindilaurate (50 uL) were added in order. The reaction was conducted at 70 °C for 10 h. Finally, a catalytic amount of hydrochloric acid was added with vigorous stirring. Notably, the mixture gradually gelled. After dried at 60 °C for 24 h, the gel was cut into small pieces. It was then further dried at 40 °C in vacuo to the constant weight, and the SPE product was obtained. To avoid moisture absorption, the electric conductivity was measured with the specimens which were just taken out from vacuum oven.

## 3. Results and Discussion

### 3.1. Synthesis of PHU-PEO-PSSQ Networks

**Scheme 1:**
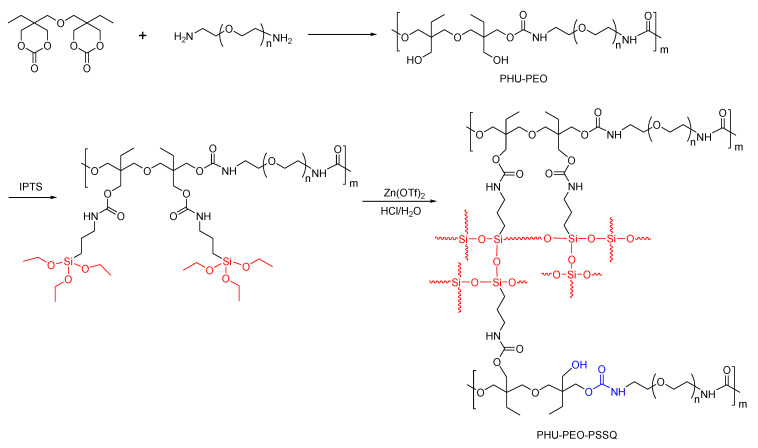
Synthesis of PHU-PEO-PSSQ networks.

**Figure 1:**
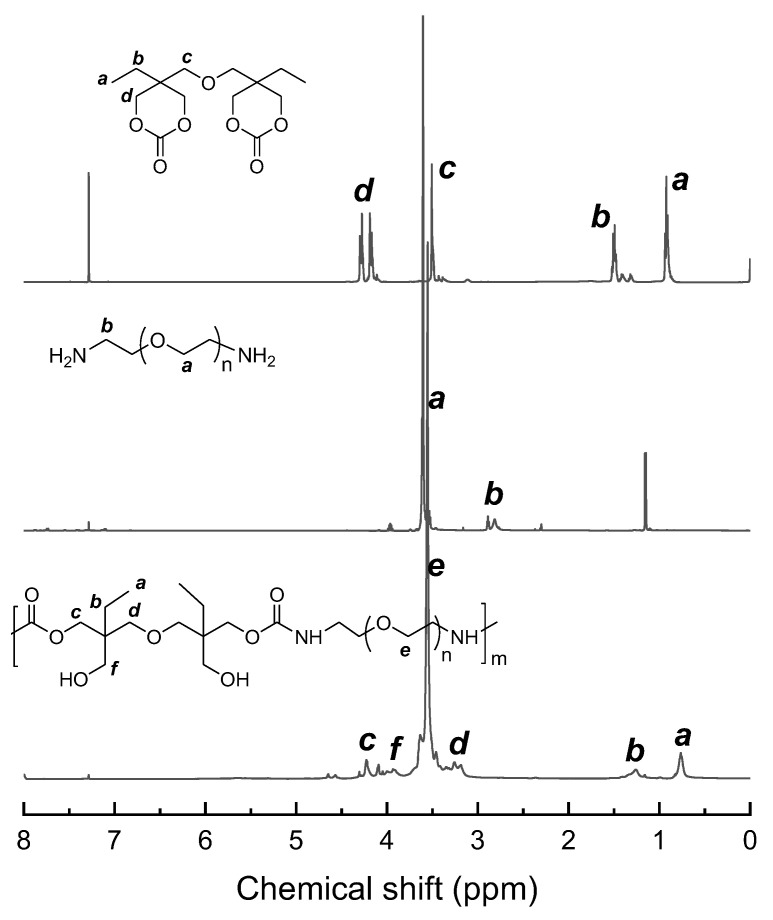
^1^H NMR spectra of B6CC, NH_2_-PEO-NH_2_ and PHU-PEO_1000_.

**Figure 2:**
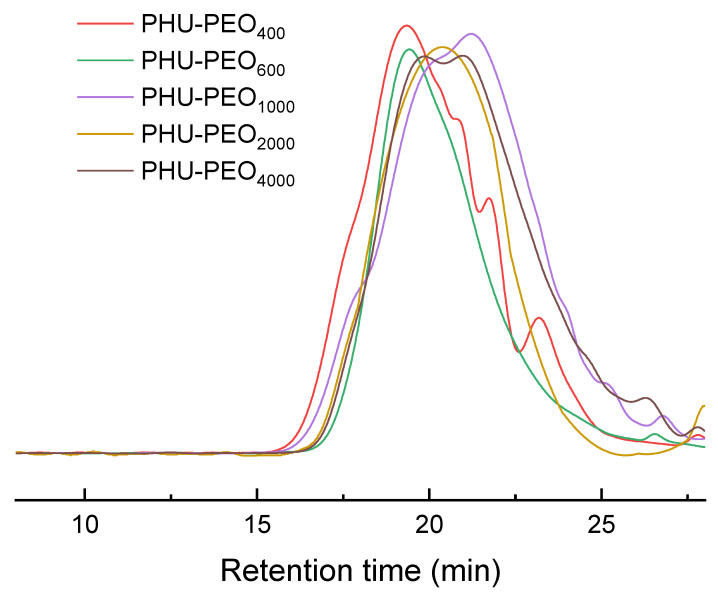
GPC curves of PHU-PEO multiblock copolymers.

The route of synthesis for the PHU-PEO-PSSQ networks is shown in Scheme 1. First, linear polyhydroxyurethane-poly(ethylene oxide) (PHU-PEO) multiblock copolymers were synthesized via the step-growth polymerization of B6CC with the equimolar NH_2_-PEO-NH_2_; the latter were synthesized via a multi-step reaction with poly(ethylene glycol)s (PEGs) as the starting materials [51], as detailed in SI. In this work, the PEGs were used with the molecular weights of *M*_n_ = 400, 600, 1000, 2000 and 4000 Da, respectively; the PHU-PEO multiblock copolymers were thus synthesized with variable contents of PEO. Through the reaction of the side hydroxyl group of PHU with isocyanate group of 3-isocyanatopropyltriethoxysilane (IPTS), triethoxysilane groups were grafted onto the main chains of PHU-PEO copolymers. The hydrolysis and condensation of these triethoxysilane groups led to the generation of PHU-PEO-PSSQ networks, in which polysilsesquioxane (PSSQ) segments were the crosslinking links. Representatively displayed in Figure 1 are the ^1^H NMR spectra of B6CC, NH_2_-PEO_1000_-NH_2_ and PHU-PEO_1000_. For B6CC, the resonance signals at 4.16~4.72, 3.50, and 1.48 ppm were attributed to the protons of methylene groups, whereas 0.89 ppm was attributed to the protons of methyl groups. For NH_2_-PEO_1000_-NH_2_, the resonance signals at 2.82 and 3.61 ppm were attributed to the protons of methylene groups of the repeating units. The former corresponds to the protons of methylene connected to amino groups whereas the latter to those in the middle of PEO chains. After the polymerization with B6CC, the signals of resonance at 4.22, 3.95 and 3.22 ppm were displayed, and assigned to the protons of methylene groups from the ring opening of B6CC. The PHU-PEO multiblock copolymers were further subjected to gel permeation chromatography (GPC). All of the samples displayed the sufficiently high molecular weights of *M_n_* = 22,100~32,400 Da with *M*_w_*/M*_n_ = 1.32~1.53 (Figure 2). The ^1^H NMR and GPC results indicate that the linear PHU-PEO multiblock copolymers were successfully synthesized. 

The linear PHU-PEO multiblock copolymers were used to react with IPTS to afford the networks. Upon adding IPTS and a catalytic amount of hydrochloric acid, notably, the DMF solutions of PHU-PEO copolymer gradually gelled, suggesting the generation of crosslinking (See Figure S1). The crosslinking resulted from the hydrolysis and condensation of triethoxysilane moieties, which were connected to the PHU-PEO main chains. The PHU-PEO-PSSQ networks were subjected to Fourier transform infrared (FTIR) spectroscopy. Representatively displayed in Figure S2 are the FTIR spectra of PHU-PEO_1000_ multiblock copolymer and its network crosslinked with PSSQ (denoted PHU-PEO_1000_-PSSQ). Compared to the former, there appeared a new band at 840 cm^−1^. This band is assignable to the stretching vibration of the C-Si bond, indicating that 3-isocyanatopropyltriethoxysilane was introduced. In the meantime, the band at 1638 cm^−1^ was intensified. This band is assignable to the stretching vibration of carbonyl of carbamate moiety. The increase in intensity for the band at 1638 cm^−1^ is ascribed to the reaction of isocyanate with hydroxyl groups, i.e., the number of carbamate moieties increased with the reaction. In addition, the band in the range of 1200~975 cm^−1^ was significantly broadened with the occurrence of the reaction. The band broadening is responsible for the overlap of the stretching vibration of Si-O-Si linkage with that of ether oxygen bonds (C-O-C). Of these two bands, the Si-O-Si linkages were generated by hydrolysis and condensation of triethoxysilane moieties. The FTIR spectroscopy indicates that PSSQ segment was successfully created and acted as the crosslinker of PHU-PEO copolymers. 

**Figure 3:**
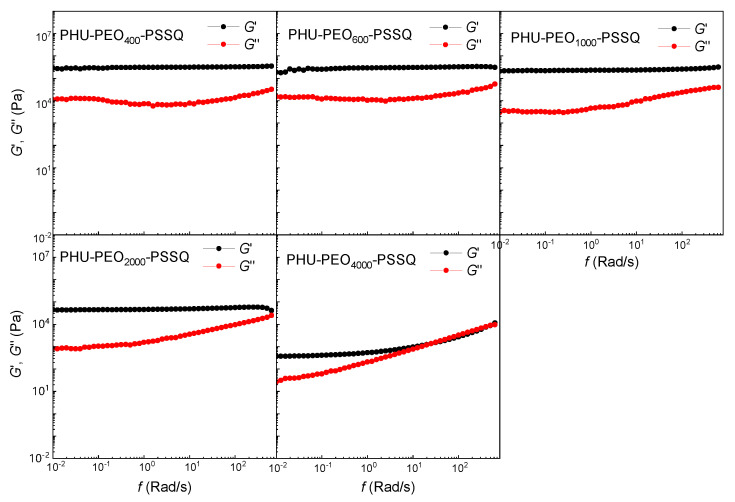
Rheological data of PHU-PEO-PSSQ networks with SAOS measurements.

The crosslinking of the PHU-PEO multiblock copolymers was studied by rheological analysis; the rheological measurements were conducted at 60 °C, a temperature above the melting points of PEO and the glass transition temperatures (*T*_g_’s) of the networks. In this work, the small amplitude oscillation shear (SAOS) measurements were performed; the rheological data are presented in Figure S3. For all of the linear PHU-PEO copolymers, the dynamic shear moduli (*G*′ and *G*″) increased with the shear frequency rising, indicating that these linear copolymers displayed typical viscoelastic properties. It is seen that the *G*″ curves were above the *G*′ curves. This observation indicates that these PHU-PEO multiblock copolymers behaved as the viscoelastic liquids. Notably, while 3-isocyanatopropyltriethoxysilane was introduced and the hydrolysis and condensation were carried out, the *G*′ values exceeded the *G*″ values (Figure 3), indicating that the samples were transformed into the elastic solids. In other word, the crosslinked networks were generated by hydrolysis and condensation. Notably, the shorter the PEO chains, the higher the *G*′ values, indicating that the crosslinking densities increased while the lengths of PEO chains decreased. Interestingly, the *G*′ curves gradually became flat, irrespective of shear frequency, especially for the samples with the PEO blocks shorter than *M*_n_ = 4000 Da. This observation suggests that the PHU-PEO-PSSQ networks exhibited the behavior of perfect elastomeric solids. The above results indicate that the linear PHU-PEO copolymers have been successfully crosslinked with PSSQ. 

**Figure 4:**
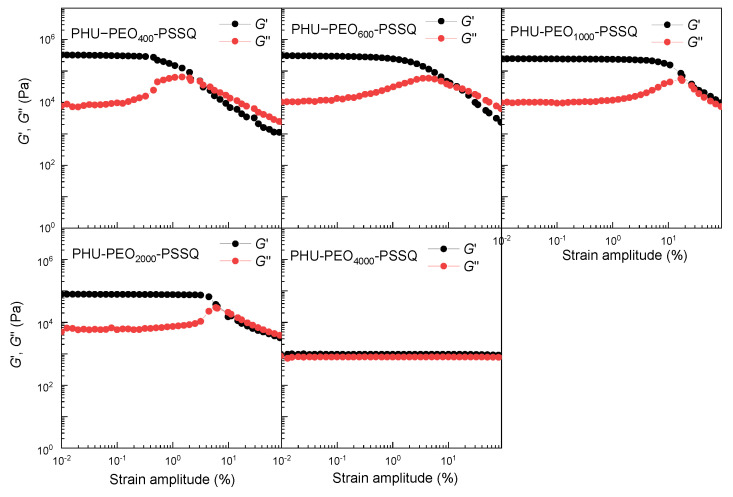
Rheological data of PHU-PEO-PSSQ networks with LAOS measurements.

A further insight into the nature of PHU-PEO-PSSQ networks can be obtained with the large amplitude oscillation shear (LAOS) measurements. For the LAOS measurements, the shear frequency was set at *f* = 10 rad/s with variable strain amplitude from *γ* = 10^−2^~10^2^%. Figure 4 shows the plots of dynamic moduli (*G*′ and *G*″) versus strain amplitudes. All the samples displayed the strain softening phenomena except for the PHU-PEO_4000_-PSSQ network, suggesting that the network structures via chemical crosslinking can be destructed while the strain amplitude was sufficiently large. It is proposed that the strain softening is attributable to the occurrence of the exchange of silyl ether bonds in the presence of Zn(OTf)_2_ [52,53]. For PHU-PEO_4000_-PSSQ network, the linear viscoelastic region was observed at the strain amplitude from 10^−2^ to 10^2^%, suggesting that the dynamic exchange of silyl ether bonds was fairly weak since the content of PSSQ was fairly low. Increasing the contents of PSSQ (or decreasing the lengths of PEO chains) resulted in the strain softening appearing at low strain amplitudes. For the PHU-PEO_2000_-PSSQ network, the strain softening was detected at *γ* = 5.8%. For the PHU-PEO_400_-PSSQ network, the linear viscoelastic region was narrowed to *γ* = 0.33%, suggesting that the silyl ether metathesis can occur at a low strain amplitude. Except for PHU-PEO_4000_-PSSQ network, all other networks had the intersection points between the *G*′ and *G*″ curves. This observation suggests that these PHU-PEO-PSSQ networks had the plasticity in solids, i.e., the networks can be reprocessed. According to the theories of rubber elasticity, the crosslinking densities of networks (ν) can be estimated from the dynamic storage moduli of the linear viscoelastic regions with the following Equation [54]:

(1) $$\upsilon =\frac{G^{\prime }}{RT}$$

where *G*′ is the shear storage modulus in the linear viscoelastic regions as shown in Figure 4; *T* was temperature and *R* was the gas constant. The crosslinking densities were calculated and listed in Table S1. Notably, the crosslinking densities increased with decreasing the lengths of PEO chains. 

### 3.2. Thermomechanical Properties

**Figure 5:**
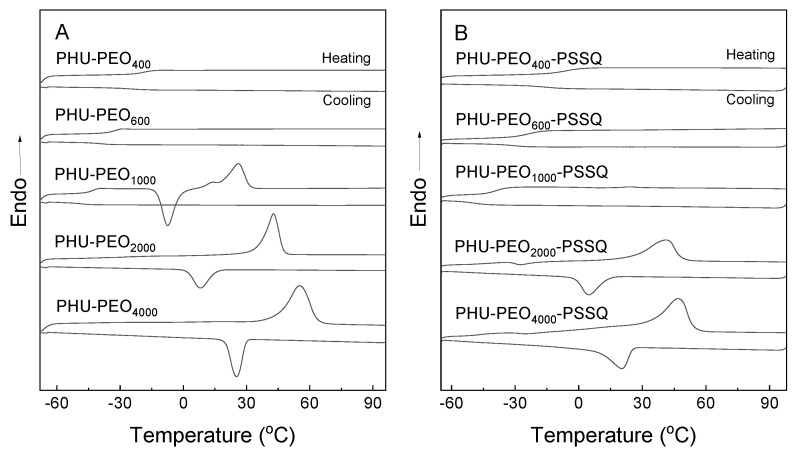
DSC curves of: (**A**) PHU-PEO multiblock copolymers; (**B**) PHU-PEO-PSSQ networks.

All PHU-PEO-PSSQ networks were subjected to differential scanning calorimetry (DSC). For comparison, the linear PHU-PEO multiblock copolymers were also measured under the same condition. Figure 5A shows the DSC curves. For all of the samples, the single glass transitions were displayed in the range of −50 to −20 °C. It is seen that the *T*_g_’s were highly dependent on the lengths of PEO chains. The shorter the PEO chains, the higher the *T*_g_’s. The single and composition-dependent *T*_g_ behavior indicates that the linear PHU-PEO copolymers had the homogenous amorphous phases. For the PHU-PEO copolymers with the PEO lengths of *L*_PEO_ = 1000 Da or higher, there were the endothermic and exothermic peaks in the heating and cooling scans, respectively. The former is ascribed to the melting of PEO crystals whereas the latter to the crystallization of PEO chains. Notably, the longer the PEO chains, the higher the *T*_m_’s and *T*_c_’s of PEO blocks. The increase in *T*_m_’s is readily explained on the basis of the increase in perfection of crystals with increasing the lengths of PEO (denoted *L*_PEO_) whereas the increase in *T*_c_’s is accounted for the increase in difficulty of PEO crystallization, as the lengths of PEO chains were decreased. In the cooling scans, notably, only the samples with *L*_PEO_ = 2000 Da or longer displayed crystallization, indicating that the crystallization of PEO chains was significantly suppressed with the generation of the homogenous amorphous phase. Upon crosslinking, notably, the crystallization was further suppressed (Figure 5B). It was noteworthy that the melting and crystallization transitions were only detected for the networks with *L*_PEO_ = 2000 Da or longer; the *T*_m_’s were further decreased, suggesting that the generation of crosslinking significantly suppressed the crystallization of PEO chains. 

**Figure 6:**
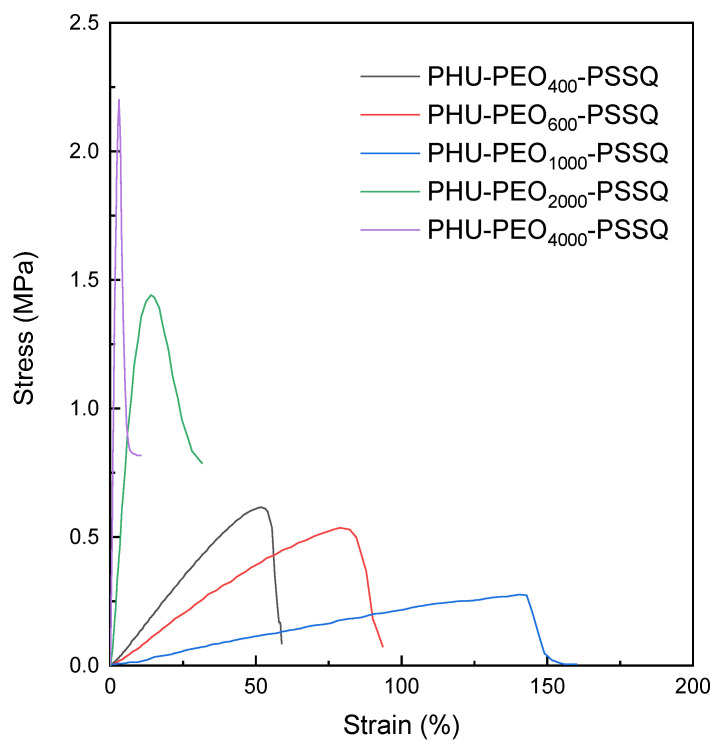
Stress–strain curves of PHU-PEO-PSSQ networks.

The mechanical properties of the PHU-PEO-PSSQ networks were measured with the tensile mechanical tests. At room temperature, PHU-PEO_400_-PSSQ, PHU-PEO_600_-PSSQ and PHU-PEO_1000_-PSSQ exhibited the stress–strain curves typical of elastomers (Figure 6). With the increasing PEO lengths, the Young’s modulus and tensile strength decreased, whereas the elongation at break was increased. For PHU-PEO_400_-PSSQ network, the Young’s modulus and tensile strength were measured to be *E* = 1.02 MPa and *σ*_b_ = 0.62 MPa, respectively. For PHU-PEO_1000_-PSSQ, the E and *σ*_b_ values were decreased to 0.21 and 0.28 MPa, respectively. Nonetheless, the elongation at break (*ε*_b_) was increased from c.a. 52 to 142%. The increased *ε*_b_ values suggest that the PHU-PEO-PSSQ networks became increasingly ductile as the contents of PEO segments increased. Notably, the *E* and *σ*_b_ values significantly increased and the *ε*_b_ values decreased while the length of PEO segments was *L*_PEO_ = 2000 Da or higher (Table S1). This observation is ascribed to the reinforcement of PEO crystals. 

### 3.3. Reprocessing Properties

**Figure 7:**
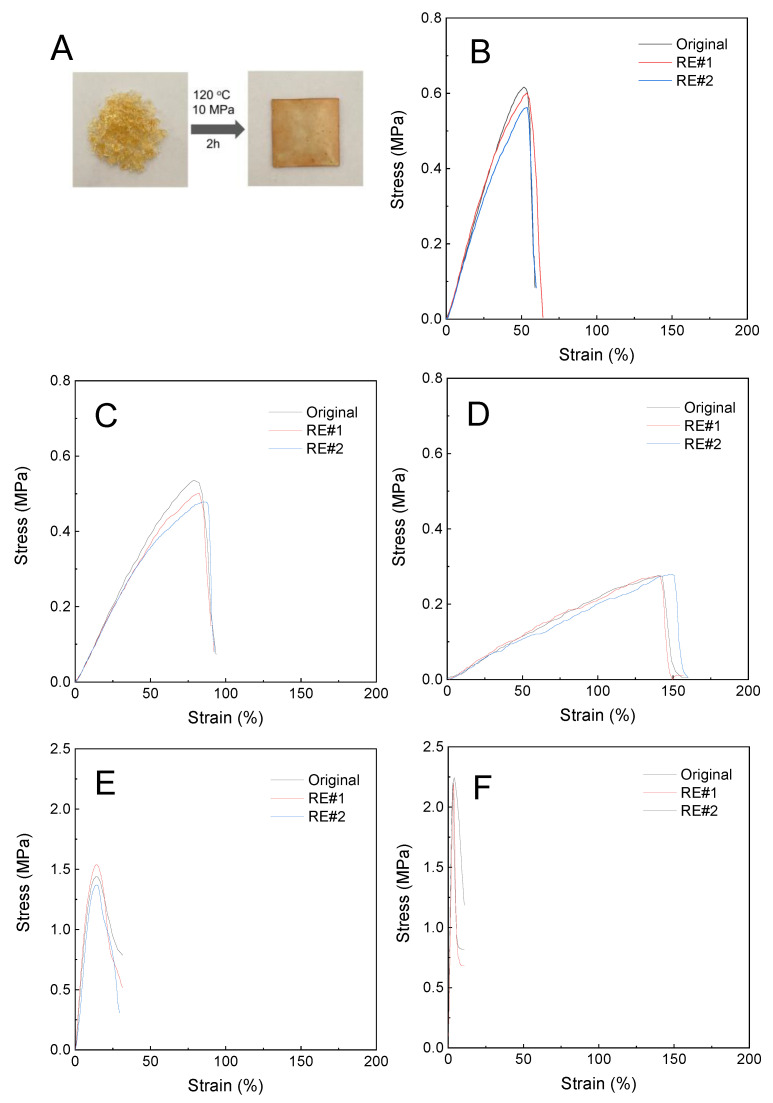
Reprocessing behavior of PHU-PEO-PSSQ networks: (**A**) Photos of PHU-PEO_1000_-PSSQ networks after and before reprocessing; (**B**) the stress–strain curves of PHU-PEO_400_-PSSQ, (**C**) PHU-PEO_600_-PSSQ, (**D**) PHU-PEO_1000_-PSSQ, (**E**) PHU-PEO_2000_-PSSQ and (**F**) PHU-PEO_4000_-PSSQ networks with different numbers of reprocessing treatments.

**Figure 8:**
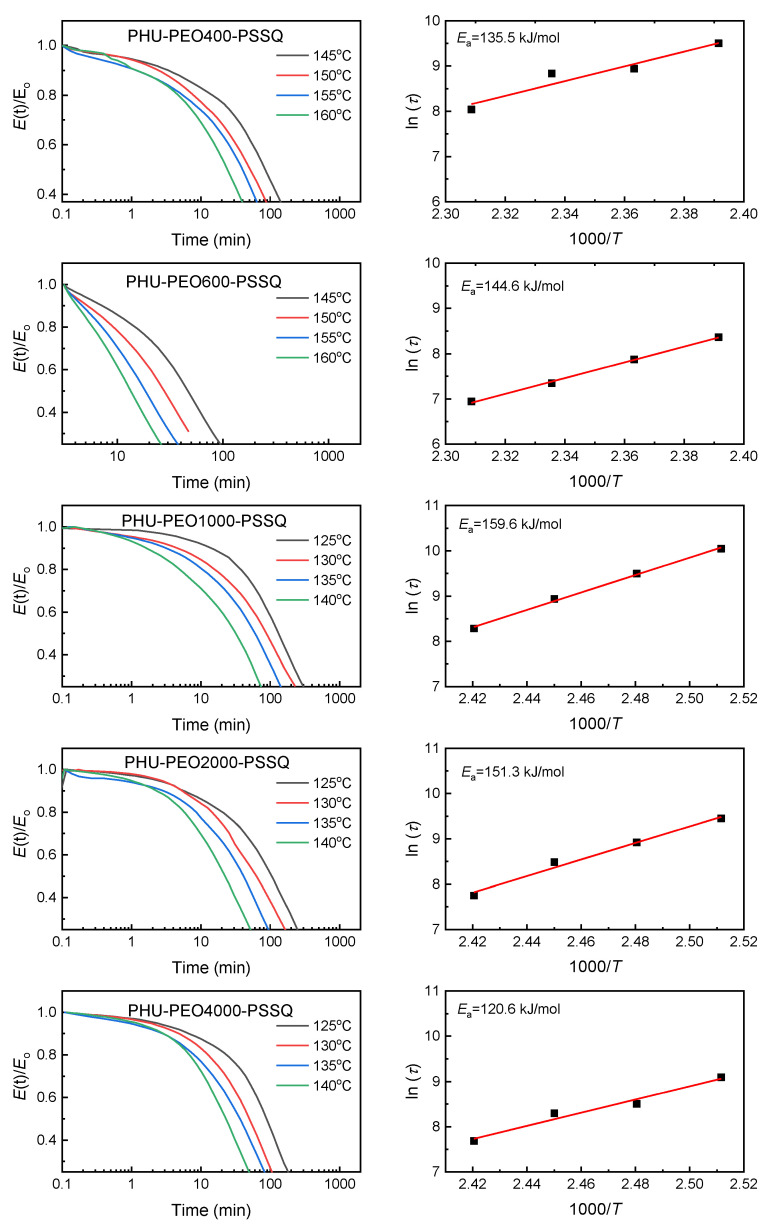
Stress relaxation curves of PHU-PEO-PSSQ networks: (**Left**) Stress relaxation curves; (**right**) plots of ln (*τ*) as functions of 1/*T* for the determination of apparent activation energy (*E*_a_) values.

Returning to Figure 4, the LAOS tests showed that the PHU-PEO-PSSQ networks had the reprocessing properties in the presence of Zn(OTf)_2_. The reprocessing properties were further demonstrated with the following reprocessing tests. Taking the PHU-PEO_1000_-PSSQ network for instance, the sample was first cropped into small pieces. The cut samples were then pressed at 120 °C. Notably, these small pieces were successfully integrated into a sheet without discernable flaws (See Figure 7A), indicating that the PHU-PEO-PSSQ networks can indeed be reprocessed. To examine the recovery of mechanical properties, the tensile tests were carried out. For the reprocessed specimens, the stress–strain curves are presented in in Figure 7B–F. Notably, the stress–strain curves were almost overlapped with those of the original specimens, i.e., the mechanical properties were almost fully recovered. This result indicates that the PHU-PEO-PSSQ networks can indeed be reprocessed. It is proposed that in the presence of Zn(OTf)_2_, the PHU-PEO-PSSQ networks were reshuffled via the metathesis of silyl ether bonds of PSSQ linkages. It should be pointed out that the transesterification of carbamates with side hydroxyl groups of PHU could additionally be involved with the rearrangement of the PHU-PEO-PSSQ networks at an elevated temperature (~120 °C). To examine this situation, we prepared the PHU-PEO-PSSQ networks without introducing Zn(OTf)_2_. Under an identical condition, notably, we failed to obtain the sheet without defects (See Figure S4). The specimen could not be welded into a monolithic sheet until it was further hot-pressed at 140 °C, suggesting that the transesterification of carbamates did not ensure the reprocessing of the networks at 120 °C. In the meantime, the PHU-PEO_1000_-PSSQ networks with and without Zn(OTf)_2_ were subjected to stress relaxation tests; the curve of stress relaxation is shown in Figure S5. Notably, these two specimens exhibited quite different relaxation curves, suggesting that the dynamic chemistries involved with both of the samples were quite different. With an identical time, notably, the relaxation of the network without Zn(OTf)_2_ was much slower than that with Zn(OTf)_2_. To investigate the effect of the PEO lengths (or contents) on the dynamic exchanges, the stress relaxation tests were carried out for all PHU-PEO-PSSQ networks. Figure 8 shows the stress relaxation curves of the PHU-PEO-PSSQ networks. In all the cases, the stresses can be well relaxed, suggesting that these networks can be indeed reshuffled. Considering that there were two types of dynamic chemistries (i.e., silyl ether metathesis and carbamate transesterification), we extracted the stress relaxation times (*τ’s*) by using the Kohlrausch–Williams–Watts (KWW) stretched exponential decay function instead of the Maxwell model, which is based on single exponential decay of stress. The KWW function [55,56] is written as below:

(2) $$\frac{E(t)}{E_{o}}=\exp\left[-{\left(\frac{t}{\tau *}\right)}^{\beta }\right]$$

where *E*(*t*)/*E_o_* is the normalized modulus at time (*t*); τ* stands for the characteristic time; β represents the stretching exponent characterizing the breadth of relaxation time distribution; the *β* value is between 0 and 1. For *β* = 1, the relaxation is in a single exponent manner. By non-linear curve fitting, we can obtain the τ* and *β* values (Tables S4–S8), which can be used to determine the average relaxation time (*τ*) using the following equation [57]:

(3) τ=τ*Γ(1β)β

where Γ is the gamma function. In this work, we fit the data of stress relaxation tests (Figure 8). The curve fitting is quite well, yielding the average relaxation times (*τ*) (Tables S4–S8). By using the *τ* values, we calculated the apparent activation energy (*E*_a_) values of the dynamic exchanges in the system with Arrhenius equation [58]:

(4) $$\tau =Ae^{\frac{-E_{a}}{RT}}$$

where *A* and *R* are the pre-exponential factor and gas constant. For PHU-PEO_400_-PSSQ, PHU-PEO_600_-PSSQ, PHU-PEO_1000_-PSSQ, PHU-PEO_2000_-PSSQ and PHU-PEO_4000_-PSSQ, the *E*_a_’s were determined to be 135.6, 144.6, 159.6, 151.3 and 120.6 kJ·mol^−1^, respectively. Notably, the *E*_a_ values displayed the maximum (i.e., *E*_a_ = 159.6 kJ·mol^−1^) for the PHU-PEO_1000_-PSSQ network. It is proposed that there are two opposite factors that affect the *E*_a_ values. On the one hand, the *E*_a_ values were affected by the crosslinking densities since the crosslinked structures constitute the restriction of the dynamic exchange reactions. The higher the crosslinking densities, the more difficult the dynamic exchange reactions, the higher the *E*_a_ values. In this system, the PHU-PEO_400_-PSSQ network had the highest crosslinking densities among these samples. On the other hand, the dynamic exchange reactions (i.e., the sily ether metathesis and carbamate transesterification) were influenced by the continuity of hydroxyurethane and PSSQ structural units. For the network with the short PEO chains, the structural units remained contiguous, facilitating the dynamic exchanges. With increasing the PEO lengths, the structural units gradually became disconnected or isolated. The discontinuity of hydroxyurethane and PSSQ structural units was unfavorable to the dynamic exchanges. The longer the PEO chains, the more difficult the dynamic exchanges, the higher the *E*_a_ values. It is the combination of the above opposite factors that result that PHU-PEO_1000_-PSSQ network had the highest *E*_a_ values among these PHU-PEO-PSSQ networks. 

### 3.4. Polyelectrolyte Properties 

**Figure 9:**
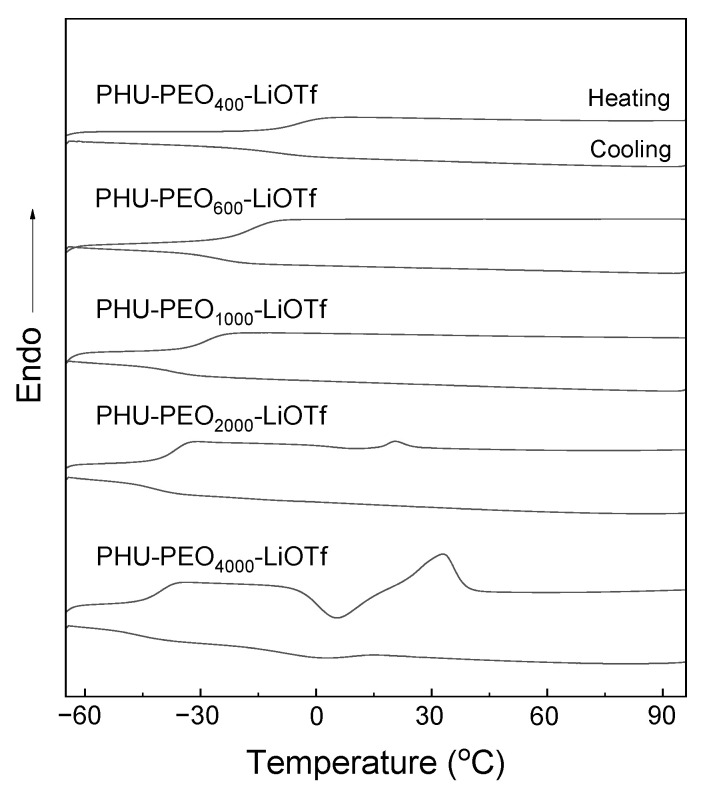
DSC curves of PHU−PEO−LiOTf SPEs.

**Figure 10:**
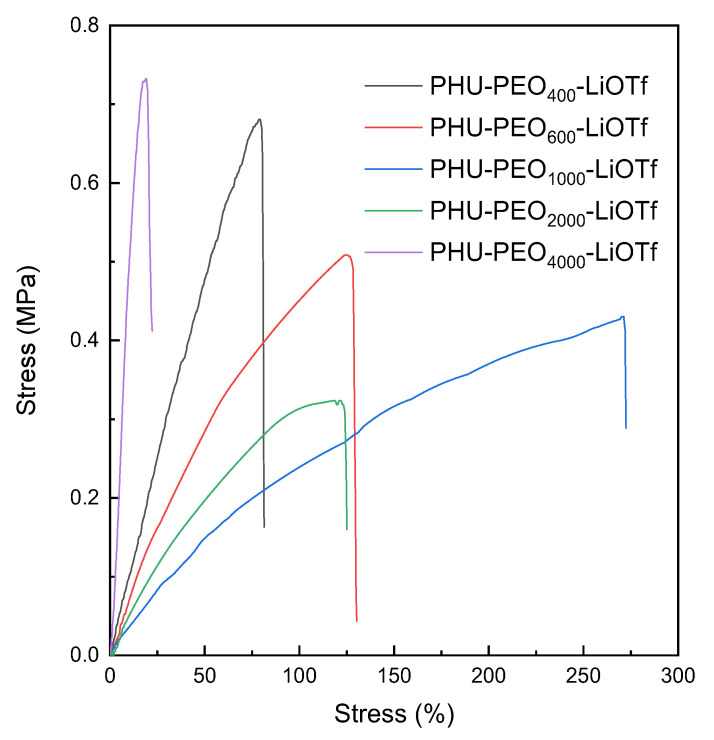
Stress–strain curves of PHU−PEO−LiOTf SPEs.

The low *T*_g_’s and low crystallinity of the PEO-based networks remind to prepare the solid polyelectrolytes (SPEs) by incorporating electrolyte. In this work, lithium trifluoromethyl sulfonate (LiOTf) was introduced into the PHU-PEO-PSSQ networks by controlling the molar ratio of the salt to ethylene oxide (EO) to be 1:12. In all the cases, notably, the homogenous and transparent composites were obtained, suggesting that no macroscopic phase separation occurred. Shown in Figure 9 are the DSC curves of the PHU-PEO-LiOTf SPEs. For the SPEs with the length of PEO segments lower than 1000 Da, no endothermic transitions assignable to PEO were detected, suggesting that the PEO chains in SPEs were non-crystallizable. Compared to the networks without LiOTf, the crystallization was also weakened while the lengths of PEO were *L*_PEO_ = 2000 Da or higher. The fact that the PEO chains no longer crystallized is ascribed to: (i) the restriction of network structure on the crystallization and (ii) the complexation of PEO segments with Li^+^ ions. The introduction of LiOTf also significantly affected the mechanical properties of the materials. Figure 10 shows the stress–strain curves of the composites. For the SPEs with relatively short PEO chains (i.e., *L*_PEO_ = 400, 600 and 1000 Da), the Young’s modulus and tensile strengths were higher than those of the networks without LiOTf (See Table S2). The improved mechanical strengths are attributable to the increase in the inter-component interactions since the complexation of PEO with Li^+^ ions have been introduced. Owing to the complexation, the crystallization of PEO chains was further suppressed while the lengths of PEO were *L*_PEO_ = 2000 Da or higher. As a consequence, the Young’s moduli and strength at break would be slightly decreased due to the decrease in crystallinity of PEO. For the SPEs with the lengths of PEO over *L*_PEO_ = 4000 Da, the Young’s moduli and strength at break were indeed lower than those of the networks without the salt, but the elongation at break were slightly increased (See Table S2).

**Figure 11:**
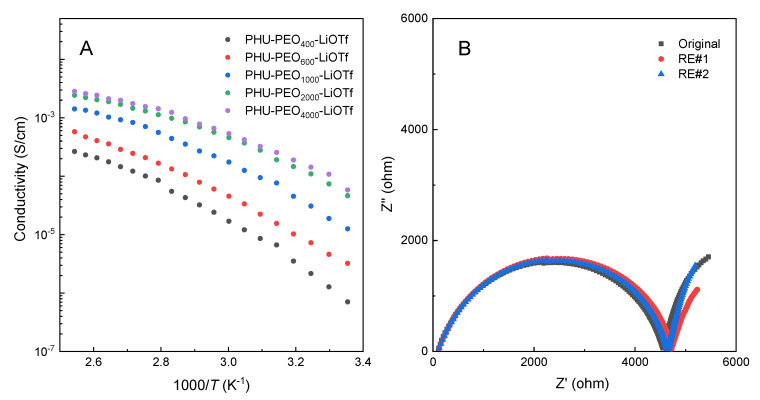
Ionic conductivity of: (**A**) PHU−PEO−LiOTf SPEs at variable temperature; (**B**) Nyquist plots of PHU-PEO_1000_-LiOTf before and after reprocessing.

All of the PHU-PEO-LiOTf SPEs were then subjected to the measurements of ionic conductivity by means of impedance analysis in the temperature range from 25 to 120 °C. Figure 11A displayed the plots of ionic electric conductivity (σ) with reciprocal of temperature (1/*T*). Notably, the ionic conductivity increased with the temperature rising. Furthermore, the σ values increased with the lengths of PEO chains. At the identical temperature, the longer the PEO chains, the higher the σ values. At 300 K, the σ values were measured to be 9.13 × 10^−7^, 3.79 × 10^−6^, 1.50 × 10^−5^, 5.62 × 10^−5^ and 7.64 × 10^−5^ S × cm^−1^ for the SPEs containing the PEO length of 400, 600, 1000, 2000 and 4000, respectively. For PHU-PEO_1000_-LiOTf, PHU-PEO_2000_-LiOTf and PHU-PEO_4000_-LiOTf, notably, the quite high σ values were obtained, which makes SPEs the candidates for application in lithium battery. It is proposed that the ionic conductivity was dependent on the crystallinity of PEO and the crosslinking densities of the networks. The lower the crystallinity, the lower the crosslinking density, and the higher the σ values. For PHU-PEO-LiOTf SPEs, the crystallization of PEO chains was restricted with: (i) the generation of networks crosslinked with PSSQ and (ii) the complexation of Li^+^ cations with PEO. The PHU-PEO-PSSQ network structures could exert two opposite influences on the ionic conductivity. On the one hand, the crosslinked networks suppressed the crystallization of PEO, which is favorable to the increase in the *σ* values. On the other hand, the crosslinked networks constituted the restriction of the transport of Li^+^ and OTf^-^ ions, which is unfavorable to the increase in the *σ* values. 

It is worth noticing that the relationship of ionic conductivity with temperature did not follow the Arrhenius equation. Nonetheless, it can be well depicted with Vogel–Tamman–Fulcher (VTF) equation [59,60,61,62]:

(5) $$\sigma =AT^{-1/2}\exp[-B/R(T-T_{0})]$$

where *A*, *B* and *T*_o_ are the pre-exponential factor, pseudo-activation energy and the equilibrium glass transition temperature, respectively. This observation suggests that the ionic conductivity was related to the ionic hopping motion, which was coupled with the relaxation/breathing and/or segmental motion of polymer chains. In Equation (5), *B* was interpreted as the apparent activation energy required to provide suitable free volume for ion conduction [63]. Through non-linear curve fitting with Equation (5), we obtained the *A* and *B* values, which are summarized in Table 1. Notably, the *B* values were dependent on the lengths (or contents) of PEO segments, displaying the minimum (viz. *B* = 5.82 KJ·mol^−1^) for PHU-PEO_4000_-LiOTf. It is proposed that the *B* values were affected by two opposite factors. On the one hand, the *B* values increased with the crosslinking densities rising since the crosslinked structures had the restriction on the ionic transport. The higher the crosslinking densities, the lower the electric conductivity. In this case, the crosslinking densities decreased with increasing the length of the PEO chains. As a consequence, the *B* values displayed a declining tendency with the increase in the PEO lengths. On the other hands, the *B* values would be significantly affected by the crystallinity of PE chains. The generation of PEO crystals would restrict the ionic transport in the SPEs. The longer the PEO chains, the stronger the tendency of PEO crystallization. The increase in crystallinity of PEO chains would result in the increase in *B* values. Therefore, the apparent activation energy (*B*) values reflected the combined contribution of the above two factors (See Table 1). Notably, the *B* values displayed a monotonous decrease with the increment of the PEO lengths, suggesting that the impact of PEO crystallite was small. In fact, the DSC results showed that the crystallization of PEO was indeed significantly suppressed (Figure 9). It is proposed that the *A* value in Equation (5) is proportional to the quantities of effective charge carriers. The generation of PEO crystallization and the increased crosslinking densities of the networks would significantly restrict the transport of Li^+^ ions. 

**Figure 12:**
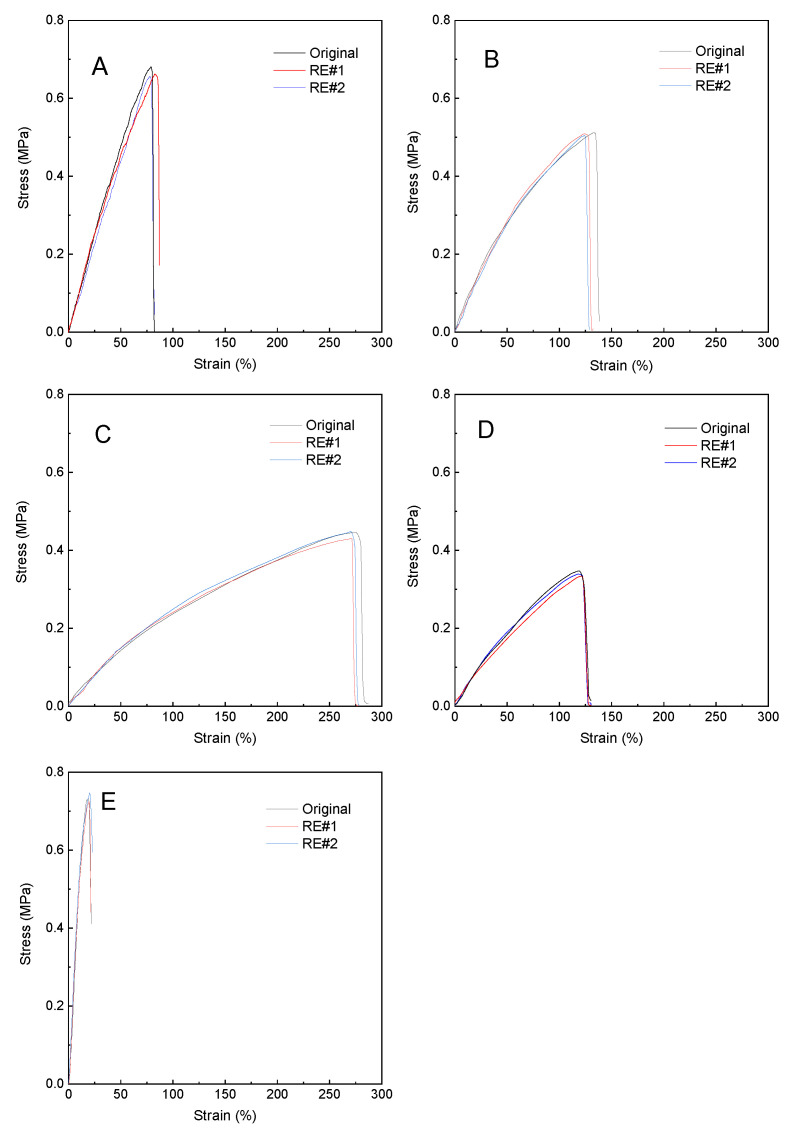
Stress–strain curves of: (**A**) PHU-PEO_400_-LiOTf, (**B**) PHU-PEO_600_-LiOTf, (**C**) PHU-PEO_1000_-LiOTf, (**D**) PHU-PEO_2000_-LiOTf and (**E**) PHU-PEO_4000_-LiOTf for reprocessing tests.

It is highly of interest to investigate the impact of the reprocessing properties of the PHU-PEO-LiOTf SPEs on the ionic conductivity while they were used as solid polyelectrolytes. In this work, the solid polyelectrolyte samples were cut into small pieces, which were then hot-pressed into compact ribbons. The reprocessed samples were used for the measurements of the mechanical properties and ionic conductivity. Figure 12 shows the stress–strain curves of the solid polyelectrolyte samples before and after reprocessing. The stress–strain curves of the reprocessed samples were almost the same as the original ones, suggesting their excellent reprocessing properties. At the same time, the ionic conductivity was measured for the reprocessed (or recycled) samples. Figure 11B shows the Nyquist plots for PHU-PEO_1000_-LiOTf, which was reprocessed twice. The curves of the initial and reprocessed samples almost coincided with each other. The results indicate that the reprocessing did not affect the transport of ions in the composite, i.e., no defects were created with the reprocessing. The reprocessing properties suggest that the SPEs are recyclable. 

## 4. Conclusions

A series of PHU-PEO multiblock copolymers were synthesized via the step-growth polymerization of B6CC with NH_2_-PEO-NH_2_. These multiblock copolymers were reacted with 3-isocyanatopropyltriethoxysilane and the reacted products were crosslinked via hydrolysis and condensation of triethoxysilane moieties. By using NH_2_-PEO-NH_2_ with various molecular weights, we obtained the PHU-PEO-PSSQ networks with variable contents of PEO. The PHU-PEO-PSSQ networks displayed the mechanical properties dependent on the contents of PEO. It was found that the PHU-PEO networks displayed excellent reprocessing properties in the presence of trifluoromethanesulfonate [Zn(OTf)_2_]. Compared to the PHU networks crosslinked via the reaction of difunctional cyclic carbonate with multifunctional amines, the organic-inorganic PHU networks displayed the decreased reprocessing temperature. Further, we transformed this crosslinked networks into the solid polymer electrolytes by introducing lithium trifluoromethyl sulfonate (LiOTf). The formation of crosslinking networks and the complexation of PEO and Li^+^ ion endowed the materials with relatively high ionic conductivity. The ionic conductivity was as high as 7.64 × 10^−5^ S × cm^−1^ at 300 K. Moreover, the solid polymer electrolytes were reprocessable and the reprocessing behavior did not affect the ionic conductivity. 

## Figures and Tables

**Table 1:** Parameters of Vogel–Tamman–Fulcher (VTF) equation.

Samples	*T*_o_ (*K*)	*A* (SK^1/2^ cm^−1^)	*B* (kJ mol^−1^)
PHU-PEO_400_-LiOTf	201.94	2.91 × 10^−3^	8.51
PHU-PEO_600_-LiOTf	202.42	5.65 × 10^−3^	8.41
PHU-PEO_1000_-LiOTf	197.56	6.31 × 10^−3^	7.19
PHU-PEO_2000_-LiOTf	195.35	4.60 × 10^−3^	5.89
PHU-PEO_4000_-LiOTf	194.80	4.44 × 10^−3^	5.82

## Data Availability

Data are contained within the article and supplementary materials.

## Supplementary Materials

The following supporting information can be downloaded at: https://www.mdpi.com/article/10.3390/polym15244634/s1. Scheme S1: Synthetic route of NH_2_-PEO-NH_2_; Table S1: Crosslinking densities of PHU-PEO-PSSQ networks; Table S2: Mechanical properties of PHU-PEO-PSSQ networks; Table S3: Mechanical properties of PHU-PEO-LiOTf SPEs; Table S4: The KWW function parameters and average relaxation times obtained from best fits to the PHU-PEO400-PSSQ stress relaxation data; Table S5: The KWW function parameters and average relaxation times obtained from best fits to the PHU-PEO600-PSSQ stress relaxation data; Table S6: The KWW function parameters and average relaxation times obtained from best fits to the PHU-PEO1000-PSSQ stress relaxation data; Table S7: The KWW function parameters and average relaxation times obtained from best fits to the PHU-PEO2000-PSSQ stress relaxation data; Table S8: The KWW function parameters and average relaxation times obtained from best fits to the PHU-PEO4000-PSSQ stress relaxation data; Figure S1: The photos of the mixture of PHU-PEO and IPTS before and after hydrolysis and condensation; Figure S2: FTIR spectra of PHU-PEO_1000_ and PHU-PEO_1000_-PSSQ; Figure S3: Rheological data of PHU-PEO multiblock copolymers; Figure S4: The reprocessing process of PHU-PEO_1000_-PSSQ network (A) with, and (B) without (ZnOTf)_2_ as catalyst; Figure S5: The stress relaxation curves of PHU-PEO_1000_-PSSQ networks with, and without (ZnOTf)_2_.
