# Supplementary material for: Polyhydroxyurethane and Poly(ethylene oxide) Multiblock Copolymer Networks: Crosslinking with Polysilsesquioxane, Reprocessing and Solid Polyelectrolyte Properties
Source: Polymers (Basel). 2023 Dec 7;15(24):4634. doi: 10.3390/polym15244634 (PMC10747941; doi:10.3390/polym15244634)
Supplement: Supplementary file 1 [file polymers-15-04634-s001.zip › polymers-2753952-supplementary.pdf]

Supporting Information

S1. EXPERIMENTAL

S1.1. Materials

Poly(ethylene glycol)s with the molecular weights of M_n = 400, 600, 1000, 2000 and 4000) were purchased from Adamas Reagent Co, Shanghai, China. *p*-Toluenesulfonyl chloride, potassium phthalimide, hydrazine hydrate, di(trimethylolpropane), triethylamine and ethyl chloroformate were supplied by Adamas Reagent Co, Shanghai, China. The organic solvents, such as dichloromethane (DCM), 1,4-dioxane and N,N'-dimethylformamide (DMF), petroleum ether, ethanol and chloroform, were obtained from Shanghai Titan Scientific Co, Ltd.

S1.2 Synthesis of α,ω-diamino poly(ethylene oxide) (NH_2-PEO-NH_2)

In a flask, *p*-toluenesulfonyl chloride (19.400 g, 0.102 mol) and triethylamine (12 mL) were added to a solution of PEO1000 (10.000 g, 10 mmol) in chloroform (60 mL), and the mixture was stirred at room temperature overnight. The polymer precipitated by the addition of cold diethyl ether to the mixture was collected by filtration and dried under vacuum to yield ditosylated-PEO. A mixture of ditosylated-PEO (5.500 g, 5.5 mmol) and potassium phthalimide (33.500 g, 185 mmol) in DMF (80 mL) was refluxed under a nitrogen atmosphere for 12 h. The precipitate formed was then filtered. Diethyl ether was added to the transparent filtrate. The precipitate was filtered and dried under vacuum to yield phthalimide-PEO. A mixture of phthalimide-PEO (5.000 g, 5 mmol) and hydrazine hydrate (30.0 mL, 0.8 mol) in ethanol (120 mL) was refluxed for 20 h. After filtering the insoluble materials, the solution was concentrated and then precipitated into diethyl ether. After drying, amine-terminated PEO were obtained with a yield of 65%. ^{1}H NMR (ppm, $CDCl_3$): 2.82 (*s*, 2H, -C$\boldsymbol{H}_2$-NH_2), 3.61 (-CH_2-CH_2-O-).

S1.3 Synthesis of bis(6-membered cyclic carbonate) (B6CC)

In a three neck round bottom flask, di(trimethylolpropane) (20.000 g, 80.0 mmol) was dispersed in dry tetrahydrofuran (320 mL) and triethylamine (24.400 g, 240 mmol) was added in suspension drop by drop at 0 °C. Ethyl chloroformate (25.000 g, 230 mmol) was added at 0 °C and the reaction was performed for 12 h at room temperature. After the reaction, salt was removed by filtration and THF was removed by rotary evaporator to obtain a white solid which was further recrystallized in THF. The white crystals of bicyclic six membered carbonate (18.350 g, 76.2 % yield) was obtained. ^{1}H NMR (ppm, $CDCl_3$): 0.89 (*s*, 6H, C$\boldsymbol{H}_3$), 1.48 (*q*, 4H, -CH_3CH_2C-), 3.50 (*s*, 4H, -CH_2OCH_2-), 4.16 ~ 4.72 (*d*, 4H, C$\boldsymbol{H}_2$OCOOCC$\boldsymbol{H}_2$-).

S2. MEASUREMENTS AND TECHNIQUES

S2.1 Nuclear Magnetic Resonance (NMR) Spectroscopy

The ^{1}H NMR spectra were recorded with a Bruker Advance 500 spectrometer at room temperature. Deuterium chloroform was used as the solvent.

S2.2 Gel Permeation Chromatography (GPC)

The molecular weights of polymers were measured on a Waters 1515 HPLC system equipped with three Waters RH columns (RH1, 3, 4) and a RI detector. N,N-dimethylformamide (DMF) containing 0.01 M lithium bromide (LiBr) was used as the eluent at the elution ration of 1.0 mL × min^{-1}. The molecular weights were expressed with polystyrene standards.

S2.3. Fourier Transform Infrared (FTIR) Spectroscopy

The FTIR measurements were conducted on a Perkin Elmer Paragon 1000 Fourier transform spectrometer at room temperature (25 °C). The films of specimens were

obtained via casting the chloroform solution of the samples (2 wt%) onto KBr windows. For the preparation of PHU-PEO-PPSQ networks, the samples were compressed into ultrathin transparent films for measurements. All the specimens were sufficiently thin to be within a range where the Beer–Lambert law is obeyed. In all cases 64 scans at a resolution of 2 cm^{-1} were used to record the spectra.

S2.4. Differential Scanning Calorimetry (DSC)

The DSC measurements were performed on a TA Instruments Q2000 differential scanning calorimeter in a dry nitrogen atmosphere. A heating rate of 20 °C × min^{-1} and cooling rate of 10 °C × min^{-1} were used to record the thermograms. Before the measurements, the samples were heated up to 150 ^{o}C and held at this temperature for 3 min and then quenched to -70 ^{o}C to erase the thermal history.

S2.5 Mechanical Tests

The stress–strain tests were carried on WDW-2 universal electron testing machine (Songdun Instruments Co. Ltd., Shanghai, China) at room temperature. Rectangular specimens were prepared and a loading rate of 100 mm × min^{-1} was used.

S2.6 Rheological Measurements

The rheological measurements were performed on a DHR-2 stress-controlled rheometer (TA, USA) at 60 ^{o}C. The circular samples with the diameter of 2 cm were used for the measurement. For the large amplitude oscillation shear (LAOS) measurements, the shear frequency was set at f =10 rad/s with variable strain amplitude of γ= 10^{-2} ~ 10^{2} %. For the small amplitude oscillation shear (SAOS) measurements, the strain was set at 0.1% with variable shear frequency of 0.01~100 rad/s.

S2.7 Ionic conductivity test

The impedance plot was performed on a Microtest 6630 impedance analyzer. For this purpose, the specimens with 1.0 mm thickness and 15.0 mm diameter were machined with the frequency ranging from 10 Hz to 100 kHz. All the measurements were carried out with the 0.5 V of alternating current across the two opposite sides of a film specimen. The samples were kept in a temperature-controlled oven. The ionic conductivity at different temperature was calculated by the following equation:

$$\sigma = \frac{d}{R \times S} \tag{S1}$$

where d and S represent the thickness and area of the samples, respectively. R denotes the bulk resistance of the samples which can be calculated by measuring the Nyquist plots.

S3. SCHEME

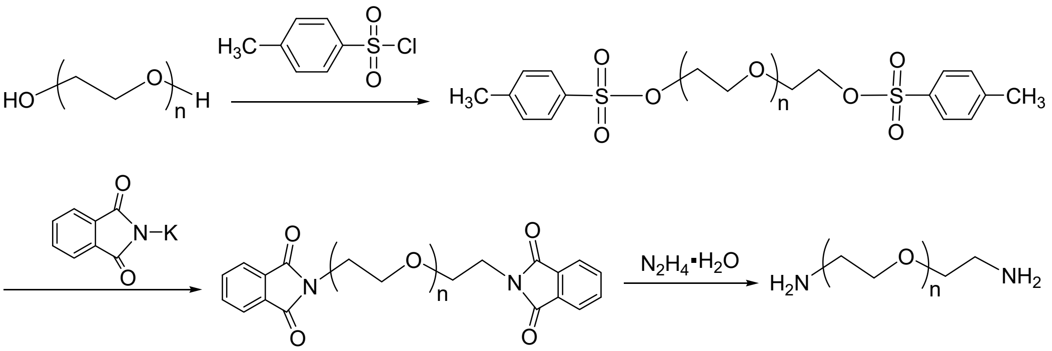

Scheme S1. Synthetic route of NH_2-PEO-NH_2.

S4. TABLES

Table S1. Crosslinking densities of PHU-PEO-PSSQ networks.

Samples	T(K)	G' (Pa)	υ(mol×m^{-3})
PHU-PEO$_{400}$-PSSQ	333.15	3.02×10^5	109.03
PHU-PEO$_{600}$-PSSQ	333.15	2.90×10^5	104.70
PHU-PEO$_{1000}$-PSSQ	333.15	2.51×10^5	90.62
PHU-PEO$_{2000}$-PSSQ	333.15	0.82×10^5	29.60
PHU-PEO$_{4000}$-PSSQ	333.15	0.011×10^5	0.40

Table S2. Mechanical properties of PHU-PEO-PSSQ networks.

	Tensile strength (MPa)	Elongation at break (%)	Modulus (KPa)
PHU-PEO$_{400}$-PSSQ	0.62±0.04	53±6	1.14±0.06
PHU-PEO$_{600}$-PSSQ	0.54±0.03	81±8	0.68±0.04
PHU-PEO$_{1000}$-PSSQ	0.28±0.02	142±13	0.24±0.02
PHU-PEO$_{2000}$-PSSQ	1.45±0.06	14±2	15.6±0.15
PHU-PEO$_{4000}$-PSSQ	2.21±0.13	3±0.5	73.2±1.25

Table S3. Mechanical properties of PHU-PEO-LiOTf SPEs.

	**Tensile strength (MPa)**	**Elongation at break (%)**	**Modulus (KPa)**
PHU-PEO_{400}-LiOTf	0.67±0.06	80±3	1.15±0.12
PHU-PEO_{600}-LiOTf	0.52±0.04	125±11	0.82±0.08
PHU-PEO_{1000}-LiOTf	0.43±0.02	271±13	0.43±0.03
PHU-PEO_{2000}-LiOTf	0.32±0.04	122±6	0.34±0.02
PHU-PEO_{4000}-LiOTf	0.73±0.06	18±3	3.42±0.15

Table S4. The KWW function parameters and average relaxation times obtained from best fits to the PHU-PEO_{400}-PSSQ stress relaxation data.

T (°C)	**τ* (min)**	α	**<τ> (min)**	**R^2**
145	153.5	0.62	221.6	0.996
150	90.0	0.63	127.4	0.999
155	72.6	0.58	114.3	0.994
160	40.0	0.69	51.3	0.998

Table S5. The KWW function parameters and average relaxation times obtained from best fits to the PHU-PEO_{600}-PSSQ stress relaxation data.

T (°C)	**τ* (min)**	α	**<τ> (min)**	**R^2**
145	65.7	0.85	71.5	0.998
150	38.6	0.80	43.7	0.997
155	23.5	0.84	25.8	0.999
160	15.7	0.84	17.2	0.999

Table S6. The KWW function parameters and average relaxation times obtained from best fits to the PHU-PEO_{1000}-PSSQ stress relaxation data.

**T (°C)**	**τ* (min)**	α	**<τ> (min)**	**R^2**
125	367.5	0.91	384.4	0.999
130	204.7	0.86	221.1	0.999
135	97.3	0.68	126.7	0.998
140	51.3	0.69	65.8	0.997

Table S7. The KWW function parameters and average relaxation times obtained from best fits to the PHU-PEO$_{2000}$-PSSQ stress relaxation data.

T (°C)	τ* (min)	α	<τ> (min)	R^2
125	169.3	0.71	211.5	0.998
130	104.1	0.75	123.9	0.999
135	65.0	0.72	80.2	0.995
140	34.6	0.82	38.5	0.999

Table S8. The KWW function parameters and average relaxation times obtained from best fits to the PHU-PEO$_{4000}$-PSSQ stress relaxation data.

T (°C)	τ* (min)	α	<τ> (min)	R^2
125	134.1	0.83	148.1	0.994
130	74.7	0.84	81.9	0.999
135	56.5	0.76	66.6	0.998
140	34.3	0.89	36.3	0.999

S5. FIGURES

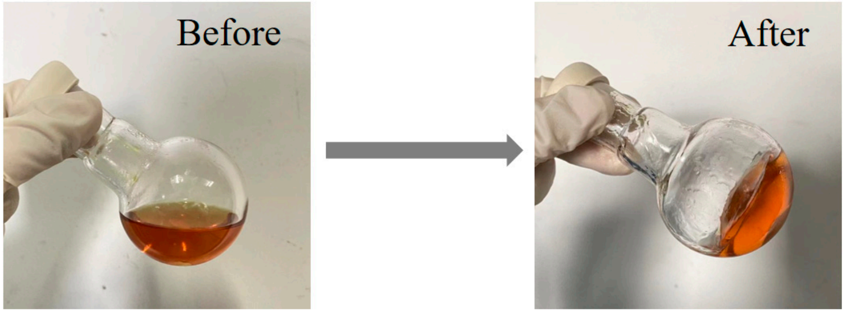

Figure S1. The photos of the mixture of PHU-PEO and IPTS before and after hydrolysis and condensation.

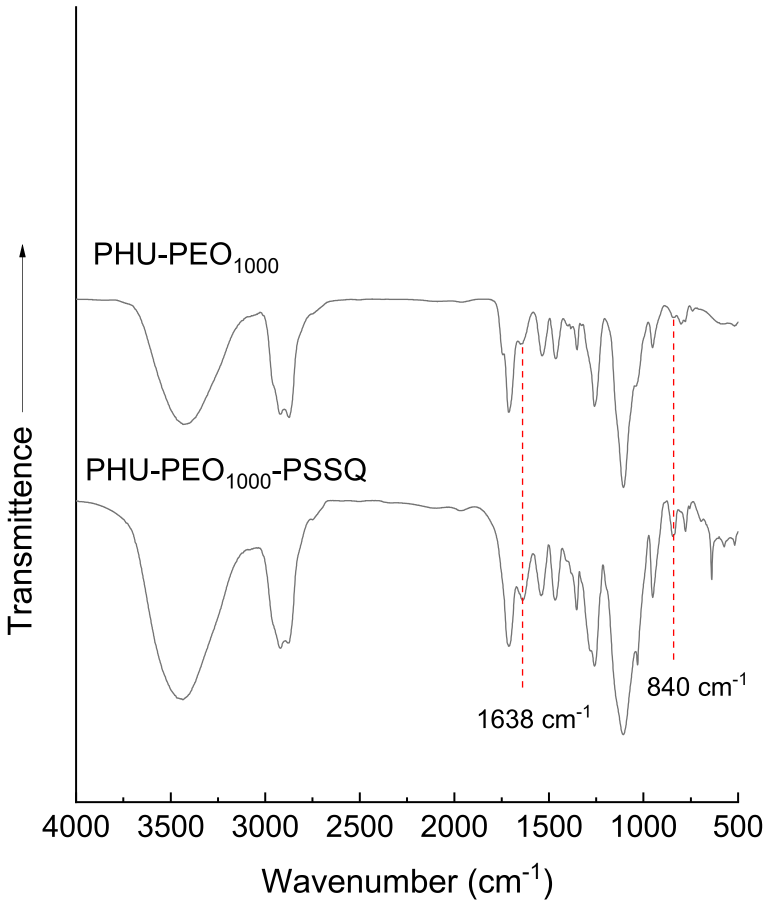

Figure S2. FTIR spectra of PHU-PEO_{1000} and PHU-PEO_{1000}-PSSQ.

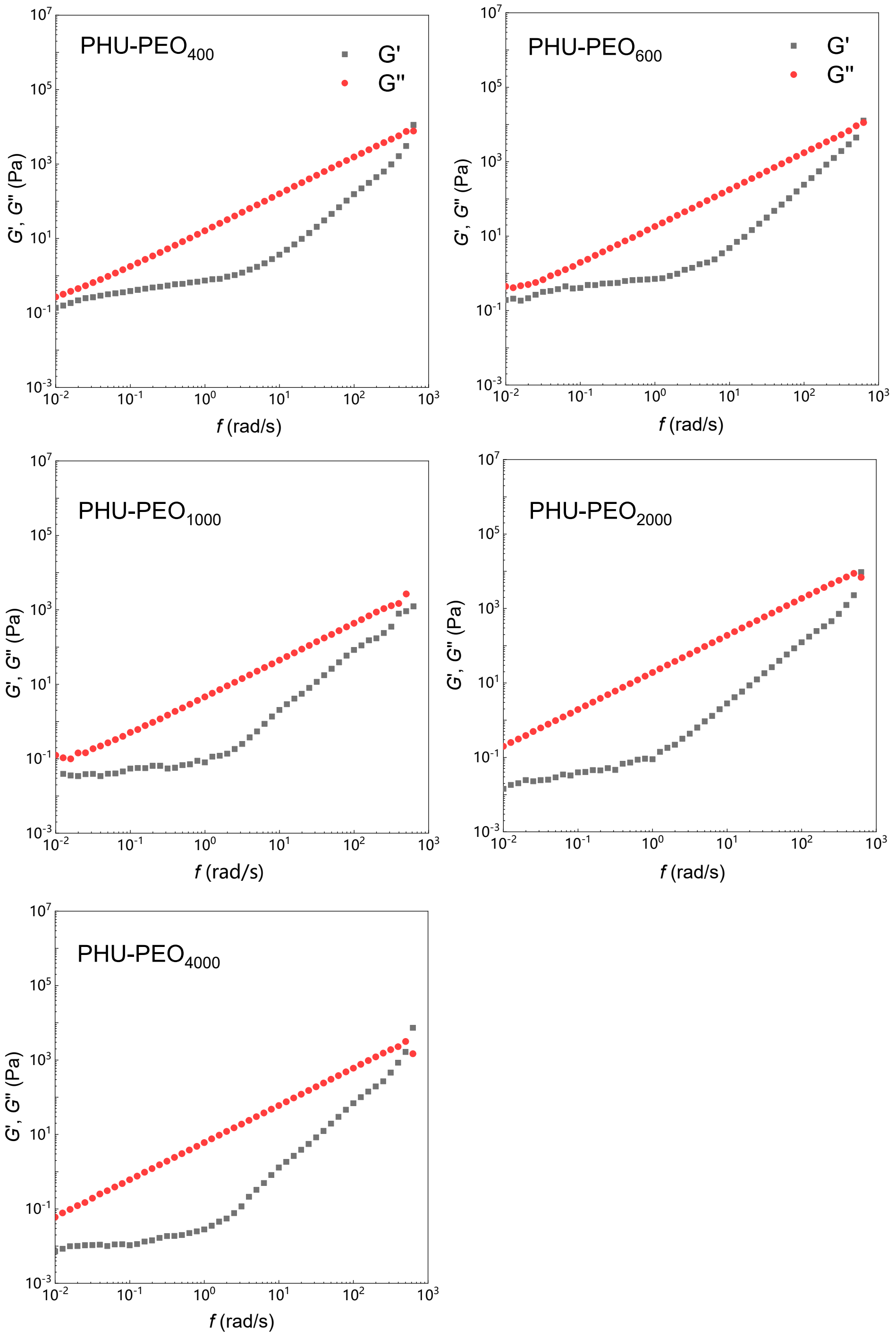

Figure S3. Rheological data of PHU-PEO multiblock copolymers.

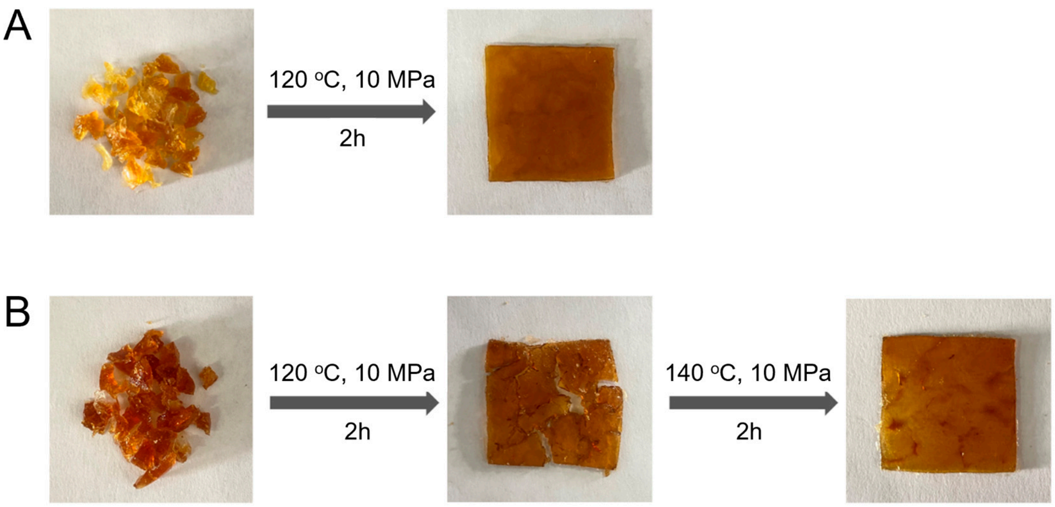

Figure S4. The reprocessing process of PHU-PEO1000-PSSQ network (A) with, and (B) without (ZnOTf)2 as catalyst.

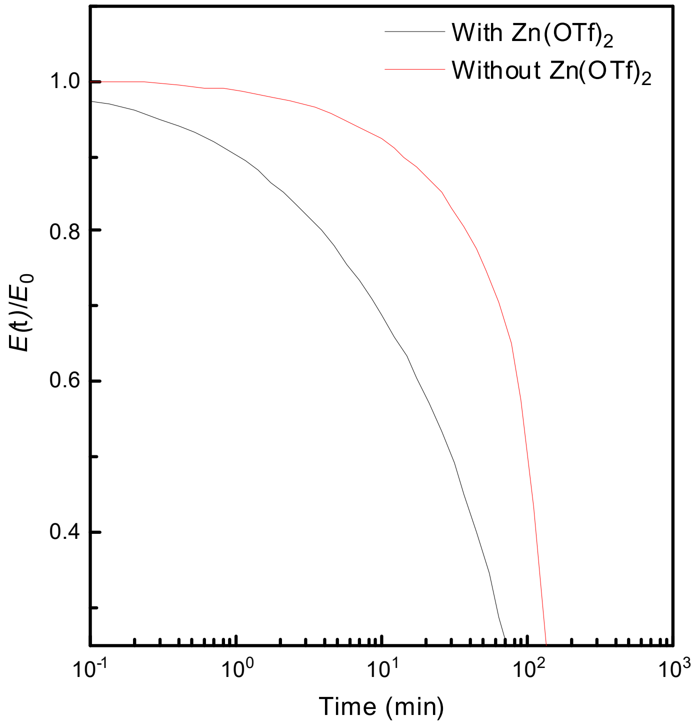

Figure S5. The stress relaxation curves of PHU-PEO1000-PSSQ networks with, and without (ZnOTf)2.
